# Favipiravir pharmacokinetics in Ebola-Infected patients of the JIKI trial reveals concentrations lower than targeted

**DOI:** 10.1371/journal.pntd.0005389

**Published:** 2017-02-23

**Authors:** Thi Huyen Tram Nguyen, Jérémie Guedj, Xavier Anglaret, Cédric Laouénan, Vincent Madelain, Anne-Marie Taburet, Sylvain Baize, Daouda Sissoko, Boris Pastorino, Anne Rodallec, Géraldine Piorkowski, Sara Carazo, Mamoudou N. Conde, Jean-Luc Gala, Joseph Akoi Bore, Caroline Carbonnelle, Frédéric Jacquot, Hervé Raoul, Denis Malvy, Xavier de Lamballerie, France Mentré, on behalf of the JIKI study group

**Affiliations:** 1 INSERM, IAME, UMR 1137, F-75018 Paris, France; Université Paris Diderot, IAME, UMR 1137, Sorbonne Paris Cité, F-75018 Paris, France; 2 Inserm, UMR 1219, Université de Bordeaux, Bordeaux, France; 3 Programme PACCI/site ANRS de Côte d’Ivoire, Abidjan, Côte d’Ivoire; 4 Assistance Publique–Hôpitaux de Paris, Hôpital Bichat Claude Bernard, Paris, France; 5 Assistance Publique–Hôpitaux de Paris, Hôpital Bicêtre, Paris, France and Inserm UMR1184, Université Paris-Sud; 6 UBIVE, Institut Pasteur, Centre International de Recherche en Infectiologie, Lyon, France; 7 Centre Hospitalier Universitaire de Bordeaux, Bordeaux, France; 8 Université Aix Marseille, Institut de Recherche pour le Développement, École des Hautes Études en Santé Publique, EPV, Marseille, France; 9 Médecins Sans Frontières Belgique, Brussels, Belgium; 10 ALIMA, Dakar, Senegal; 11 Biological Light Fieldable Laboratory for Emergencies (B-LiFE)/Belgian First Aid and Support (B-FAST), Brussels, Belgium; 12 Cliniques Universitaires Saint-Luc, Brussels, Belgium; 13 Université Catholique de Louvain, Louvain-la-Neuve, Belgium; 14 Belgian Ministry of Defense, Brussels, Belgium; 15 European Mobile Laboratory Project, Hamburg, Germany; 16 Institut National de Santé Publique, Conakry, Guinea; 17 Laboratoire des Fièvres Hémorragiques en Guinée, Université Gamal Abdel Nasser de Conakry, Conakry, Guinea; 18 Inserm, Laboratoire P4 Jean Mérieux, Lyon, France; University of Oxford, UNITED KINGDOM

## Abstract

**Background:**

In 2014–2015, we assessed favipiravir tolerance and efficacy in patients with Ebola virus (EBOV) disease (EVD) in Guinea (JIKI trial). Because the drug had never been used before for this indication and that high concentrations of the drugs were needed to achieve antiviral efficacy against EBOV, a pharmacokinetic model had been used to propose relevant dosing regimen. Here we report the favipiravir plasma concentrations that were achieved in participants in the JIKI trial and put them in perspective with the model-based targeted concentrations.

**Methods and findings:**

Pre-dose drug concentrations were collected at Day-2 and Day-4 of treatment in 66 patients of the JIKI trial and compared to those predicted by the model taking into account patient’s individual characteristics. At Day-2, the observed concentrations were slightly lower than the model predictions adjusted for patient’s characteristics (median value of 46.1 versus 54.3 μg/mL for observed and predicted concentrations, respectively, p = 0.012). However, the concentrations dropped at Day-4, which was not anticipated by the model (median values of 25.9 and 64.4 μg/mL for observed and predicted concentrations, respectively, p<10^−6^). There was no significant relationship between favipiravir concentrations and EBOV viral kinetics or mortality.

**Conclusions:**

Favipiravir plasma concentrations in the JIKI trial failed to achieve the target exposure defined before the trial. Furthermore, the drug concentration experienced an unanticipated drop between Day-2 and Day-4. The origin of this drop could be due to severe sepsis conditions and/or to intrinsic properties of favipiravir metabolism. Dose-ranging studies should be performed in healthy volunteers to assess the concentrations and the tolerance that could be achieved with high doses.

**Trial registration:**

ClinicalTrials.gov NCT02329054

## Author summary

In 2014–2015, the JIKI trial was conducted in Guinea to test favipiravir tolerance and efficacy in patients with Ebola virus disease (EDV). The main results of the trial were previously published without drug concentrations which were not available at the time of publication. The purpose of this study was to report favipiravir concentrations achieved in participants in the JIKI trial and to compare them with the targeted concentrations. We analyzed drug concentrations obtained at Day-2 and Day-4 and compared them to the targeted concentrations. At Day-2, favipiravir concentrations were significantly below but still close to the targeted concentration. At Day-4, a significant and unanticipated drop of concentrations as compared to Day-2 was observed. The origin of the lower-than-targeted concentrations and the unexpected drop could be due to severe sepsis conditions and/or to intrinsic properties of favipiravir metabolism. No significant correlation was found between the drug exposure and the virological response, indicating that it is possible that the favipiravir concentrations in the JIKI trial were not sufficient to strongly inhibit the viral replication. These findings suggest the necessity of performing dose-ranging studies with high doses of favipiravir in healthy volunteers to inform any further development of favipiravir for treatment of EVD.

## Introduction

The 2014–2016 Ebola virus disease (EVD) outbreak in West Africa has been the deadliest occurrence of the disease since its discovery in 1976. Between January 2014 and June 2016, the World Health Organization reported 28,616 EVD cases, of which 11,310 were fatal [1]. In September 2014, at the peak of the outbreak, World Health Organization launched a fast-track process to identify potential anti-Ebola drugs and established three criteria for a drug to be acceptable as a candidate for clinical trials, namely i) availability of safety data in humans ii) evidence from preclinical studies of *in vivo* efficacy against Ebola virus (EBOV) iii) sufficient drug supply.

Favipiravir, a RNA polymerase inhibitor, approved in Japan to treat non complicated influenza infection, met all three criteria [2]. First the drug demonstrated antiviral activity against EBOV both *in vitro* (with a drug EC_50_ found between 10.8 μg/mL and 63 μg/mL) and *in vivo* in mice models [3,4]. Second it had been already safely administered to more than 2000 healthy volunteers and patients worldwide [5] and its pharmacokinetics (PK) was therefore well characterized for the influenza dosing. Briefly, the drug is a small and relatively hydrophilic molecule, with a protein bound fraction of 54% and a distribution volume between 15 and 20 liters [6]. Administered orally, the drug is rapidly absorbed with a t_max_ ranging from 0.5 to 1 hour and a bioavailability close to 100% [6]. The main elimination pathway involves hepatic metabolism by aldehyde oxidase, and marginally xanthine oxidase, producing a hydrophilic and inactive metabolite M1, which is eliminated in the urine [6]. Favipiravir inhibits aldehyde oxidase, leading to time- and dose-dependent pharmacokinetics [6].

In November 2014, our group decided to perform a historically-controlled, single-arm proof-of-concept trial to assess the tolerance and efficacy of favipiravir in patients with EVD in Guinea (JIKI trial) [7]. Launching an emergency trial in the midst of such an historical outbreak posed many human, logistical, ethical and scientific challenges. Among those was the choice of the dosing regimen to be used against EBOV, which has been explained prior to the trial implementation [8]. In brief the dosing regimen was found such that it achieves safely and rapidly free average concentration comparable to that obtained in mice successfully treated while maintaining free minimal concentrations higher than the drug EC_50_. Because the pharmacokinetics is nonlinear, the search for an optimal dosing regimen was based on a pharmacokinetic model developed by the manufacturer. Using this model a dosing regimen of 1,200 mg every twelve hours was proposed for the maintenance dose, with a loading dose of 6,000 mg (2,400; 2,400; 1,200 mg) on the first day. This dosing regimen was predicted to achieve stable concentrations after 48 hours, with median total trough (pre-dose) and average concentrations in plasma of 57.0 and 83.3 μg/mL, respectively [8]. One important aspect regarding this model is that it had been developed using data collected in studies in which the highest maintenance dose received was 800 mg twice a day and the largest treatment duration was 5 days. Doses in children were derived from adult doses and adjusted for body weight [7–9].

Overall, the JIKI trial results showed that mortality was strongly associated with baseline viremia. The results provided no evidence that favipiravir monotherapy at this dose might have a favorable benefit/risk ratio in patients with very high viral load at onset, but that it would merit future research in patients with a cycle threshold (Ct) ≥ 20, corresponding to a viral load below 10^7.7^ genome copies/mL [7]. We previously published the trial results without reporting drug concentrations because they were not available at the time of the publication.

Here we report the results of the concentrations of favipiravir that were measured in patients of the JIKI trial. We compare them to the concentrations predicted by the model before the trial [8] and we analyse the possible association between drug plasma concentrations, viral loads and biochemical/haematological parameters.

## Methods

### Ethics statement

Three ethics committees were approached, namely, the institutional review board of the Institut National de la Santé et de la Recherche Médicale (Inserm, France), the Médecins Sans Frontières International Ethics Committee, and the Guinean Comité National d’Éthique pour la Recherche en Santé. All three committees commented on the protocol and approved the final version and further amendments. Even though not asked for formal approval, because it was neither the sponsor nor investigator of the study, the WHO Ethics Research Committee received the trial protocol and provided important advice that helped improve it.

### Patients

The design of the JIKI trial has been previously reported [7]. The inclusion criteria in the JIKI trial were the following: age ≥1 year, body weight ≥10kg, EVD confirmed by positive RT-PCR, no pregnancy, ability to take oral drug, oral or signed informed consent. In this PK sub-study, we included only patients of the JIKI trial that did not receive convalescent plasma prior to treatment, as we did for the main analysis [7], and who had at least one blood sample after the first day of treatment with sufficient volume to assess the favipiravir concentration.

### Treatment

All participants received standard of care and favipiravir. Favipiravir (Toyama-Chemical, 200 mg tablets) was given orally. The treatment started as soon as the consent was obtained (Day-0) and was administered for ten days. The adult dose was 6,000 mg at Day-0 (first dose: 2,400 mg; second dose eight hours after the first dose: 2,400 mg; third dose eight hours after the second dose: 1,200 mg) and 2,400 mg (1,200 mg every 12 hours) from day 1 to day 9. For children, the dose was adjusted on body weight [9].

### Drug concentration measurements

Blood samples were taken at Day-0 (baseline), Day-2, Day-4, end of symptoms, Day-14 and Day-30. Favipiravir total concentration was measured at Day-2 and Day-4 from plasma or serum samples collected less than one hour before the first favipiravir intake of the day, i.e., between 11 and 12 hours after the last drug intake.

All samples were immediately decanted. EDTA, heparin or dry tubes were divided into aliquots, frozen at -20°C, and shipped to the INSERM Jean Mérieux biosafety level 4 laboratory in Lyon. In this laboratory, they were heated at 60°C for one hour to inactivate EBOV then refrozen (-20°C) and transferred to another INSERM laboratory in Marseilles for the drug concentration measurement, using a validated procedure (**S1 Text**). Previous study on plasma samples collected in nonhuman primates has shown that the inactivation process by heating did not significantly impact the quantification of plasma favipiravir concentrations (**S2 Text**). Serum and plasma samples were analysed using the same assay technique that had been validated for plasma samples. Both types of concentrations were referred as plasma concentrations in the following.

### Virology

EBOV viraemia (molecular viral load) was immediately assessed at the onsite laboratories in four centers of the JIKI trial using a semi-quantitative RT-PCR assay (RealStar Filovirus Screen RT-PCR Kit 1.0, Altona Diagnostics). The results were expressed in terms of Ct, whose value is inversely proportional to viral load. An increase of 3 units in Ct scale corresponds approximately to a 1-log decline in viral load, therefore Ct unit corresponds to log scale for the viremia [7]. The Ct cut-off value for positivity was <40.

### Biochemistry and haematology

Biochemical and haematological parameter assays were performed using either the Piccolo Xpress (Abaxis) or the i-STAT (Abbott Laboratories) point-of-care system. We used here the results of the parameters that were available in most patients and were the most plausible to affect the drug pharmacokinetics, namely creatinine, sodium, albumin and haemoglobin.

### Mortality

All deaths during the trial were attributed to EVD and patients who were discharged were considered as patients who survived. Criteria for discharge were the absence of fever and significant symptoms for four consecutive days, ability to feed and walk independently, and two consecutive negative blood EBOV RT-PCR tests [7].

### Statistical methodology

#### Descriptive analysis of favipiravir concentrations

Descriptive statistics (median, min-max) of concentrations measured at Day-2 and Day-4 were performed. In patients having both measurements at Day-2 and Day-4, the change in concentrations was calculated. A Wilcoxon signed-rank test was performed to assess any significant change.

#### Model-based analysis for the evolution of favipiravir concentrations

In order to adjust for the variability due to individual characteristics, deviation in the dosing regimen or in the sampling times, we calculated for each observation the predicted concentration using the pharmacokinetic model provided by the manufacturer by accounting in each patient for i) the age and weight ii) the dosing regimen iii) the sampling times of drug measurements. As the drug sampling measurement was done within the hour preceding the morning dose and the exact sampling time was not reported, we assumed that blood collection was done 30 minutes before the next drug intake. The predicted concentrations on Day-2 and Day-4 were then compared to the observations at Day-2 and Day-4, respectively, using a Wilcoxon signed-rank test. In patients having concentrations at both Day-2 and Day-4, a Wilcoxon test for paired values was also used to compare the change between Day-2 and Day-4 in the predicted and the observed concentrations, respectively.

#### Drug concentrations and relationship with the virological response and mortality

Correlations between the observed concentrations at Day-2 and Day-4, respectively, and the corresponding increase in Ct value from baseline (equivalent to a decline in viral load) were tested using a Spearman rank correlation test. A Ct value above 40 was treated as equal to 40. The relationship between the observed concentrations at Day-2 and Day-4, respectively, and mortality was tested using a Wilcoxon test. Consistent with our previous findings [7], the analysis were stratified on the initial Ct value at inclusion (Ct < 20 and Ct ≥ 20), using a Benjamini-Hochberg multiple testing correction for the tests in the two groups.

#### Drug concentrations and relationship with the biochemical and haematological parameters

Correlations between the observed concentrations on Day-2 and Day-4, respectively, and the corresponding levels of biochemical or haematological parameters were tested using a Spearman rank correlation test. In patients having two concentrations, the correlation between the change in drug concentrations and the change in biochemical or haematological parameters was tested using a Spearman rank correlation test.

## Results

### Patient’s characteristics

**Fig 1:**
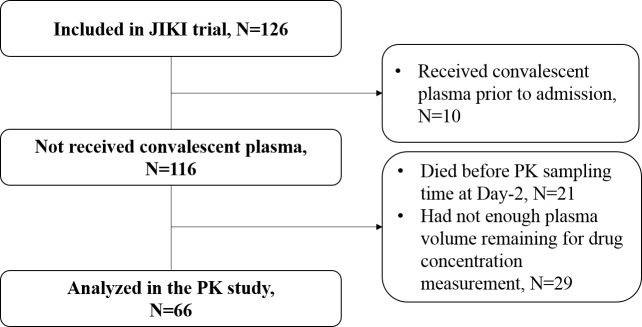
Flowchart of the patients included in the PK analysis of the JIKI trial

Of the 126 patients included in the JIKI trial, 10 were not included in the PK sub-study because they also received convalescent plasma. In addition, 21 patients died before the PK sampling time at Day-2 and 29 patients did not have enough plasma sample volume for drug concentration measurement. Thus a total of 66 patients were analysed in the PK sub-study (see flowchart in **Fig 1**), out of which 46 survived and 20 died. The median time from favipiravir initiation to death was 5 days (min-max: 2–17), with 8 patients who died between Day-2 and Day-4, 11 who died between Day-4 and Day-7 and one who died at Day-17. Patients took favipiravir as per the protocol and two missed doses were reported, both occurring the first day of drug initiation.

The characteristics of the patients included in the analysis are given in **Table 1**and are compared with those of patients included in the main analysis of the JIKI trial but not included in the PK sub-study. The patients included in this sub-PK analysis had significantly higher Ct values, lower creatinine, CRP values at baseline and significantly lower mortality rate than those who were not included (30% vs 82%, p<10^−7^). This is due in particular to the fact that the 21 patients who died before the PK sampling time at Day-2 could not be included in this sub-PK analysis (**Fig 1**).

**Table 1:** Characteristics at inclusion of the 66 patients included and the 50 patients not included in the PK analysis of the JIKI trial.

Characteristics	Patients included in PK sub-study n = 66	Patients included in JIKI trial but not included in PK sub-study and did not receive convalescent plasma n = 50	p-value^§^
**Female sex**	41 (62.1%)	28 (56.0%)	0.89
**Age**	33.5 (5–78)	28.0 (2–80)	0.07
≤ 6 y	1 (1.5%)	11 (22.0%)	
13 to 29 y	26 (39.0%)	16 (32.0%)	
≥ 30 y	39 (59.1%)	23 (46.0%)	
**Weight (kg)**	55 (14–102), 5 NA	51 (10–86), 6 NA	0.06
**Time from first symptoms to admission (days)**	4 (0–14)	3 (-1-14)	0.28
**Symptoms prior to admission**			
Fever	61 (92.4%)	44 (88.0%)	0.42
Diarrhea	35 (53.0%)	18 (36.0%)	0.07
Nausea/vomiting	30 (45.5%)	23 (46.0%)	0.95
Hemorrhage	11 (16.7%)	4 (8.0%)	0.17
Hiccup	9 (13.6%)	9 (18.0%)	0.52
Extreme fatigue	61 (92.4%)	46 (92.0%)	0.93
**Positive malaria rapid test**	8 (12.1%)	12 (24.0%)	0.09
**EBOV RT-PCR Ct value**	22.6 (14.4–32.0), 1 NA	18.1 (12.3–28.4), 5 NA	3.9x10^-7^
< 20	19 (29.2%)	32 (71.1%)	
≥ 20	46 (70.7%)	13 (28.9%)	
**EBOV viral load (log**_**10**_ **copies/mL)**	7.15 (3.40–9.23), 9 NA	8.56 (4.48–10.65), 16 NA	9.8x10^-6^
> 7.7 log_10_ copies/mL	18 (31.6%)	27 (79.4%)	
≤ 7.7 log_10_ copies/mL	39 (68.4%)	7 (20.6%)	
**Serum biochemistry**			
Creatinine (μmol/L)	114.9 (30.0–703.0), 6 NA	237.0 (31.0–1076.0), 7 NA	0.006
Creatinine < 110 μmol/L	28 (46.7%)	10 (21.3%)	
Creatinine 110 to 299 μmol/L	24 (40.0%)	16 (34.0%)	
Creatinine ≥300 μmol/L	8 (13.3%)	17 (36.2%)	
BUN (mmol/L)	7.0 (1.0–107.0), 7 NA	14.4 (1.1–60.0), 5 NA	0.002
BUN:creatinine ratio	0.06 (0.02–0.34), 7 NA	0.06 (0.03–0.27), 6 NA	0.53
Sodium (mmol/L)	132.0 (121.0–142.0), 12 NA	133.0 (124.0–143.0), 8 NA	0.1
Potassium (mmol/L)	3.9 (1.5–5.9), 13 NA	3.9 (2.5–6.7), 9 NA	0.08
Glucose (mmol/L)	5.8 (2.1–28.4), 14 NA	6.0 (1.5–14.7), 9 NA	0.71
AST (IU/L)	362.5 (28.0–2000.0), 28 NA	1008.0 (43.0–2000.0), 36 NA	0.21
ALT (IU/L)	126.0 (22.0–1356.0), 26 NA	362.5 (21.0–1698.0), 30 NA	0.03
ALT/AST ratio	0.31 (0.13–1.08), 28 NA	0.21 (0.10–0.69), 37 NA	0.04
CK (IU/L)	909.0 (109.0–5000.0), 27 NA	2038.0 (163.0–5000.0), 29 NA	0.27
Total bilirubin (μmol/L)	10.0 (0.5–19.0), 27 NA	13.0 (6.0–43.0), 30 NA	0.02
Amylase (IU/L)	92.0 (33.0–458.0), 26 NA	114.0 (24.0–434.0), 29 NA	0.66
CRP (mg/L)	15.1 (5.0–153.1), 28 NA	54.0 (11.6–200.0), 30 NA	0.0002
Albumin (g/L)	32.0 (25.0–40.0), 26 NA	29.0 (21.0–37.0), 29 NA	0.06
Haemoglobin (g/dL)	13.6 (10.2–16.7), 48 NA	15.3 (10.2–21.8), 27 NA	0.03
**Time from first symptoms to favipiravir initiation (days)**	4.5 (0–15)	4.0 (0–15)	1.0
**IV fluid compensation**	59 (89.4%)	45 (90.0%)	0.92
**Time from favipiravir initiation to death (days)**	5 (2–17)	3 (0–7)	0.0003
**Outcome**			3.4x10^-8^
Died	20 (30.3%)	41 (82.0%)	
Survived	46 (69.7%)	9 (18.0%)	

Data are in n (percent) or median (min-max).

NA: missing values.

### Children and adolescents

Among the 66 patients included in the PK sub-study, there were five adolescents (14–17 years old) and a 5-year old child. The child had a weight of 14 kg at inclusion and received 600/400/200 mg at Day-0 followed by a maintenance dose of 200 mg thrice a day. He had negative malaria test, initial EBOV Ct value of 19.9, initial EBOV viral load of 9.1 log_10_ copies/mL and no biochemical or haematological parameters before or during treatment. Three adolescents, aged 14, 14 and 17 and weighting 42, 31 and 48 kg, respectively, received loading doses of 1,600/1,600/800, 1,200/1,200/600 and 2,000/2,000/1,000 mg, respectively. Two adolescents, aged 15 and 16 years and weighting more than 50 kg received the adult dose. Among these 6 patients, only the 15-year old adolescent did not survive the infection.

### Drug concentrations

**Fig 2:**
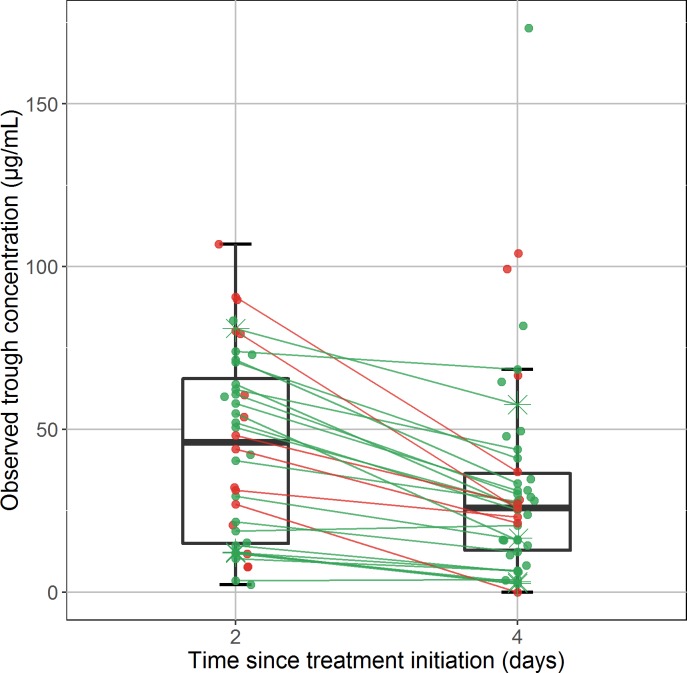
Observed trough concentrations of favipiravir at Day-2 (n = 44) and Day-4 (n = 50) after treatment initiation. Red points represent concentrations measured in patients who died during the trial, green points represent concentrations measured in those who survived. Concentrations obtained in patients receiving adult dose or weighted-based dose are presented in circles and stars, respectively. Lines connect data obtained in the 28 patients who had both measurements at Day-2 and Day-4. Boxplots represent the distribution of the concentrations. The lower and upper hinges correspond to the first and third quartiles. The upper whisker extends from the hinge to the highest value that is within 1.5 * IQR of the hinge, where IQR is the inter-quartile range, or distance between the first and third quartiles. The lower whisker extends from the hinge to the lowest value within 1.5 * IQR of the hinge.

Overall, 94 favipiravir trough concentrations were collected, 44 at Day-2 and 50 at Day-4. Among these 94 concentrations, 67 were obtained from plasma samples and 27 were from serum samples, and concentration in plasma and serum samples were largely similar (**S3 Text**). The median sampling time for the Day-2 measurement was 2.6 days after treatment initiation (min-max: 1.6–2.9) and the median sampling time for the Day-4 measurement was 4.6 days after treatment initiation (3.3–7.6). At Day-2, the median observed trough concentration was 46.1 μg/mL (23–106.9) and was close to the targeted trough concentration (57 μg/mL). However the concentrations dropped at Day-4 and the median concentration was 25.9 μg/mL (0–173.2) (**Fig 2**). This trend was observed in both plasma and serum samples (**S3 Text**). In patients having measurements at Day-2 and Day-4, the median reduction was -19.8 μg/mL (-54.6–-1.7) and was significantly different from 0 (p<10^−5^).

### Model-based analysis

**Fig 3:**
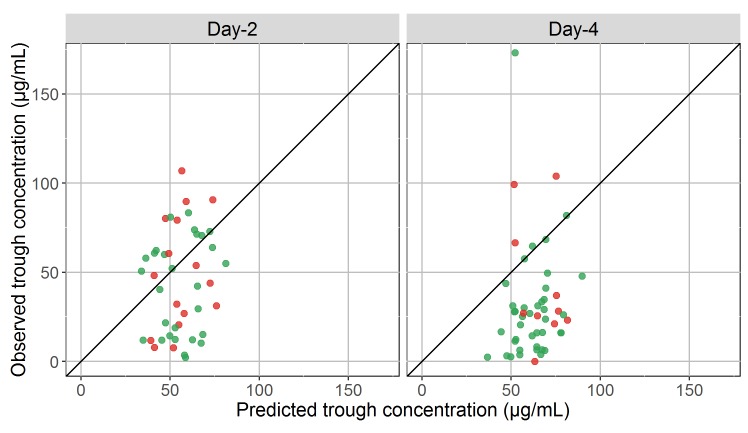
Observed trough concentrations (y-axis) versus predicted trough concentrations (x-axis) at Day-2 (left, n = 44 observations) and Day-4 (right, n = 50) after treatment initiation. Red points represent concentrations measured in patients who died during the trial, green points represent concentrations measured in those who survived.

Next, we refined our comparisons by adjusting the model’s predictions for the individual dosing regimen, sampling time, age and weight for the predicted concentrations (**Fig 3**and **Table 2**). In the 5 patients for whom the information was not available (**Table 1**), a weight of 70 kg was assumed. At Day-2 the median observed concentration was equal to 46.1 μg/mL and the median predicted concentration was equal to 54.3 μg/mL (p = 0.01). At Day-4, the difference was more pronounced, with a median observed concentration of 25.9 μg/mL compared to a median predicted concentration of 64.4 μg/mL (p<10^−6^). While the model predicted a modest median increase in concentrations equal to 5.1 μg/mL between Day-2 and Day-4, the drug concentrations actually had a marked median decrease equal to -19.8 μg/mL (p<10^−8^).

**Table 2:** Observed and adjusted predicted (from the pharmacokinetic model provided by the manufacturer) trough concentrations of favipiravir at Day-2 and Day-4 in the 66 patients included in the PK analysis of the JIKI trial.

	Number of patients	Sampling time (days after treatment initiation)	Favipiravir trough concentrations		
			Observed (μg/mL)	Adjusted Predicted (μg/mL)	p-value^§^
**Day-2**	44	2.6 (1.6–2.9)	46.1 (2.3–106.9)	54.3 (33.8–81.1)	0.012
**Day-4**	50	4.6 (3.3–7.6)	25.9 (0–173.2)	64.4 (36.7–89.7)	<10^−6^
**Day-4 minus Day-2**	28		-19.8 (-54.6–1.7)	5.1 (-3.2–26.6)	<10^−8^
**Day-4/Day-2**	28	0.54 (0–1.11)	1.08 (0.94–1.79)	<10^−7^

Data are in n or median (min-max).

^§^p-value of Wilcoxon paired test of the difference between observed and adjusted predicted concentrations.

### Relationship with virological response and mortality

**Fig 4:**
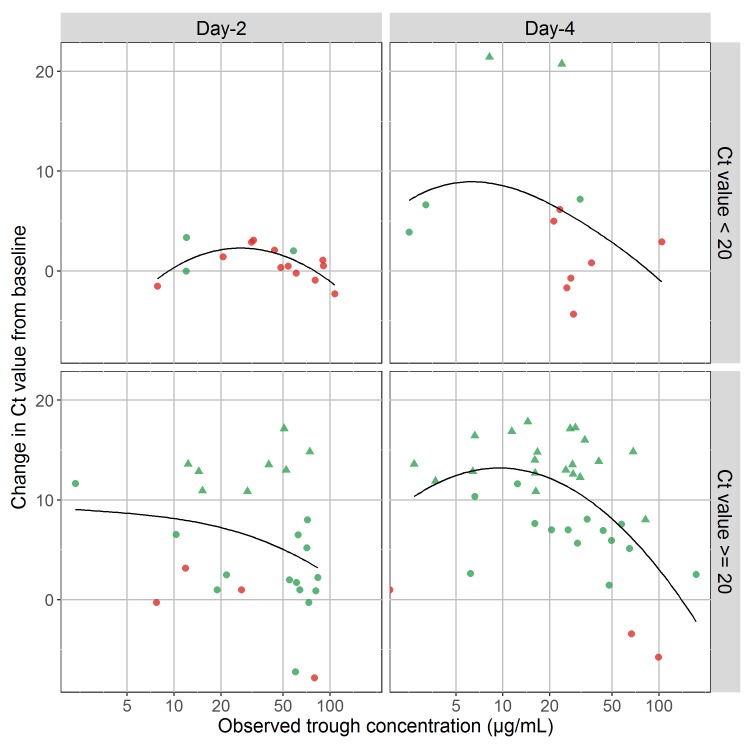
Difference in Ct values at Day-2 (left) or Day-4 (right) from baseline (the larger the value the larger the viral decline) versus drug concentrations. Top: patients with a low baseline Ct value (Ct<20) (n = 15 and 12); bottom: patients with a high baseline Ct value (Ct>=20) (bottom, n = 26 and 37). Red points represent concentrations measured in patients who died during the trial, green points represent concentrations measured in those who survived. Triangles indicate Ct values that were above 40 (detection limit) and were treated as equal to 40, and circles are observed values. Black lines are Loess trend lines.

One patient for whom no initial Ct value was available was not included in this sub-study and two PK measurements did not have corresponding Ct values. Overall 65 patients with 90 simultaneous measurements of favipiravir concentrations and Ct values were included in the analysis (41 and 49 observations at Day-2 and Day-4, respectively). Regardless of the day considered, no significant relationship between the EBOV viral decline in plasma (increase in Ct value) and favipiravir concentrations could be established (**Fig 4**) and no significant association between mortality and drug concentrations was found (**Table 3**).

**Table 3:** Observed trough concentrations of favipiravir at Day-2 and Day-4 in patients who died and those who survived according to the initial baseline EBOV viral load.

	Trough concentrations at Day-2			Trough concentrations at Day-4		
	Died	Survived	p-value^§^	Died	Survived	p-value^§^
**All patients**	41.1 (7.7–106.9), n = 16	46.4 (2.3–83.4), n = 27	0.52	27.8 (0.1–104.0), n = 10	24.6 (2.5–173.2), n = 39	0.20
**Baseline Ct value ≥ 20**	19.4 (7.7–79.3), n = 4	51.4 (2.3–83.4), n = 24	0.36	66.6 (0.1–99.3), n = 3	26.6 (2.7–173.2), n = 34	0.49
**Baseline Ct value < 20**	51.0 (7.8–106.9), n = 12	12.0 (11.9–58.0), n = 3	0.23	27.2 (21.2–104.0), n = 7	8.2 (2.5–31.2), n = 5	0.11

Data are in median (min-max).

^§^p-value calculated using Wilcoxon test to compare concentrations in patients who died and who survived. For two sub-group analysis, the threshold for significativity after Benjamini-Hochberg correction is 0.025 for the most significant p-value and 0.05 for the second p-value.

### Relationship with biochemical and haematological parameters

Longitudinal evolution of the biochemical and haematological parameters are displayed in **S1 Fig.** Albumin concentrations and haemoglobin levels decreased in most patients, while median sodium increased over time. For creatinine levels, two patterns were observed: in most patients, creatinine level decreased during treatment but in a subset of patients, creatinine increased strongly over time (**S1 Fig**). We found no significant correlation between the drug concentrations and any of the biochemical parameters at Day-2 or Day-4 (**S2 Fig**).

## Discussion

We reported here the favipiravir plasma concentrations obtained in 66 patients of the JIKI trial. The main finding of our analysis was that favipiravir concentration was significantly below but still close to the predicted value target concentration at Day-2, but decreased by nearly 50% between Day-2 and Day-4. Consequently, the concentrations at Day-4, with a median value of 25.9 μg/mL, were much below the predicted model-based value. With a protein binding of 54%, free favipiravir trough concentrations at Day-4 remained thus slightly larger than the *in vitro* EC_50_ reported in Oestereich *et al*., which was estimated at 10.5 μg/mL [4], but lower than those reported in another publication, where the EC_50_ of favipiravir was found larger than 31 μg/mL[3]. The conclusion was similar when model predictions were adjusted for individual dosing regimen or individual characteristics. In particular, these low concentrations were not due to a lack of compliance, as only two doses were not taken and both occurred on the first day of treatment initiation. Of note the assay technique was only validated for plasma samples and we did not distinguish these two types of drug concentrations. Yet serum and plasma are close matrices and similar values were observed in both plasma and serum samples, including the strong reduction in drug concentrations between day 2 and day 4 (**S3 Text**). Lastly, we did not find any significant correlation between the drug exposure and the virological response. Taken together, these results indicate that it is possible that the favipiravir concentrations in the JIKI trial were not sufficient to strongly inhibit the viral replication.

Yet this conclusion should be nuanced and taken cautiously for several reasons. First the analysis relied only on plasma favipiravir concentrations and intracellular concentrations of the active phosphorylated moiety were not available. For instance, intracellular concentrations of HIV nucleotide reverse transcriptase inhibitors were associated with antiviral efficacy, but not with the plasma concentrations of the corresponding nucleoside analogue [10]. Second our analysis only relied on pre-dose concentrations but other PK factors that could not be determined here (e.g., AUC, time above EC_50_ or EC_99_) could be a better marker of drug efficacy. Third, the fact that no significant correlation was found between the drug exposure and the virological response could also be due to the delay between infection and treatment initiation. For instance viral dynamic modelling shows that a drug affecting viral replication, such as favipiravir, will only have a limited impact on viraemia if treatment is initiated after the viraemia peak, regardless of drug efficacy [11]. Lastly, our study included only patients with drug measurements at Day-2 or after, which excluded the most severe patients who died before Day-2. Thus the patients analysed here are not representative of the JIKI population study and this is why they differed in terms of mortality, initial Ct, viral load or biochemical parameters (**Table 1**).

Two non-exclusive explanations for the lower-than-predicted concentrations and the unexpected drop between Day-2 and Day-4 can be proposed, namely the effect of the disease/treatment on the drug pharmacokinetics and the non-linear PK of favipiravir which has never been documented at this dosing regimen.

Many disease symptoms can affect the drug pharmacokinetic processes and lead to reduced concentrations. For instance reduced plasma favipiravir concentrations and altered kinetics of absorption and elimination were observed in a hamster model of arenaviral haemorrhagic fever [12]. Here in absence of frequent data points and historical data with the same dosing regimen, the effect of the disease cannot be evaluated. Obviously, the disease symptoms such as dehydration, diarrhoea, vomiting, and reduction of gut perfusion can hamper or modify favipiravir absorption. Likewise, the disease symptoms could also affect the bioavailability and the hepatic first pass, in particular through an increase in the activity of the main metabolic enzyme of favipiravir (aldehyde oxidase) with temperature [13]. In the JIKI trial, only 30 episodes of vomiting were reported within 30 minutes of drug intake, which represent 2% of the overall number of drug intakes during the trial [7]. Altered pharmacokinetics could also involve the distribution volume of favipiravir, which may be increased in Ebola patients due to treatment or to the disease itself and may explain at least in part the reduced plasma concentrations. Favipiravir’s apparent volume of distribution ranges from 15 to 20 L and is likely restricted to vascular and extra vascular fluids [5,6]. This distribution volume can be influenced by two factors, namely change in volume of body fluid and/or favipiravir protein binding. In this sub-study of the JIKI trial, 89.4% of patients received IV fluid rehydration during treatment [7], which may be responsible for haemodilution. Here, the modest decline in haemoglobin does not suggest a massive haemodilution but some vascular leakage resulting from infusion of a large volume of rehydration fluid [14] or from the disease [15] cannot be ruled out and could affect to some extent the favipiravir volume of distribution. However, such effects of infusion or disease are unlikely to solely explain the 50% reduction of favipiravir concentrations at Day-4 which would require a doubled volume of distribution. Another possible alteration of the drug PK might be due to the reduction in albumin concentrations. With a protein binding of 54%, a mean decline of about 20% in albumin levels between Day-0 and Day-4 observed in this study is unlikely to have a major effect on favipiravir distribution or elimination. Lastly, liver failure could impact favipiravir concentrations [16,17] but this should rather favour drug accumulation than accelerate elimination. Of note, no significant correlation was found between biochemical and haematological parameters and drugs concentrations but the number of observations available was limited (**S2 Fig**).

The other main cause of these lower-than-predicted concentrations could be the fact that the model used to predict the drug exposure in the JIKI trial was based on data collected in a very different context. Indeed, the drug was historically developed against influenza virus and the model was therefore developed using data collected with much lower doses of favipiravir (at most 800 mg BID) for shorter period of time (at most 5 days). Favipiravir is known to have non-linear pharmacokinetics due to its inhibitory effect on its main metabolic enzyme, aldehyde oxidase [5,6], which is also known to have several genetic polymorphisms with different catalytic activities [18]. The fact that the non-linearity of favipiravir pharmacokinetics was evaluated at doses much lower than those used in the JIKI trial [5] and that only few data on patients of African ethnicity were previously available made it complicated to predict the exposure of favipiravir with high doses in the JIKI study population. In addition, reduction in the drug concentrations over 14 days of treatment was also observed in uninfected non-human primates receiving high doses of favipiravir [19], suggesting that reduction in drug concentrations over time may be an unanticipated feature of the drug that is independent of the disease [19].

In conclusion, we have demonstrated that favipiravir plasma concentrations decreased with time and were likely too low in most patients. We advocate for a dose-ranging study on healthy volunteers to assess the pharmacokinetics and the tolerance of higher dosing regimen.

## Supporting information

S1 Text Favipiravir concentration assay. (DOCX)

S2 Text Impact of inactivation procedure on plasma favipiravir concentration. (DOCX)

S3 Text Comparison of plasma and serum concentrations. (DOCX)

S1 Fig Spaghetti plots of biochemical and haematological parameters during first week of treatment. Red points represent concentrations measured in patients who died during the trial, green points represent concentrations measured in those who survived (n = 66, 62, 43, and 23 for creatinine, sodium, albumin, haemoglobin, respectively).
(TIFF)

S2 Fig Favipiravir observed trough concentrations versus biochemical/haematological parameters at Day-2 and Day-4 after treatment initiation. Red points represent concentrations measured in patients who died during the trial, green points represent concentrations measured in those who survived. At Day-2, number of patients included in these graphs was 33, 26, 17 and 16 for creatinine, sodium, albumin and haemoglobin, respectively. At Day-4, number of patients included these graphs was 42, 36, 29 and 12 for creatinine, sodium, albumin and haemoglobin, respectively.
(TIFF)
