# Supplementary material for: Favipiravir pharmacokinetics in Ebola-Infected patients of the JIKI trial reveals concentrations lower than targeted
Source: PLoS Negl Trop Dis. 2017 Feb 23;11(2):e0005389. doi: 10.1371/journal.pntd.0005389 (PMC5340401; doi:10.1371/journal.pntd.0005389)
Supplement: S1 Text — (DOCX) [file pntd.0005389.s001.docx]

**S1 Text**

**Favipiravir concentration assay**

Favipiravir plasma total concentrations were assayed using a high performance liquid chromatography (HPLC) method with UV detection. This protocol was derived from those used in previous pharmacokinetic studies in non-human primates [1] and human [2], and was validated on plasma samples.

Briefly, 750 µL of a sodium acetate buffer (50 mmol/L) and 20 µL of internal standard, raltegravir (SIGMA) were added to a 200 µL inactivated plasma sample. The solution was vortexed (30 seconds) and centrifuged (1000 xg, 15 minutes at 4°C). For solid-liquid extraction, 750 µL of the supernatant were loaded onto a cartridge (Oasis MAX 30 µm 10 mg, Waters) pre-conditioned with 1 mL of methanol and 1 mL of ultrapure water. 300 µL of acetonitrile containing 4 vol% of formic acid were added to elute favipiravir and raltegravir. Analytes were collected in test tubes containing 50 µL of Acetonitrile containing 2 vol% of DMSO and dry evaporated under reduced pressure condition using a vacuum concentrator system (45°C, scale of rotating speed : 10 (SpeedVac, ThermoFisher)). 100 µL of ultrapure water were added to the residue, vortexed for 30 seconds and dissolved by sonication during 5 minutes. 30 µL of the samples were injected onto the HPLC system (Agilent HP series 1100) using an Inertsil ODS-3 column (4.6 mm I.D.x100 mm, 3 µm) and a guard column Inertsil ODS-3 (4.0 mm I.D.x10 mm, 3 µm). The mobile phase was composed of a 0.5 mol/L acetate TEA solution (pH 7), methanol and ultrapure water. Samples were eluted with a non-linear gradient of methanol (5-58%) and water (85-32%) at a constant flow rate (1 mL/min) for a total run time of 21 minutes. The acetate solution was eluting in constant proportion (10%). A UV detector (Agilent) was used to obtain chromatograms (360 nm). Calibration samples were analysed before each analysis in a range of concentrations from 1 to 50 µg/mL (favipiravir kindly provided by Toyama Chemical Co., Ltd and diluted in blank plasma kindly provided by the French Blood Bank (EFS)). In those conditions, favipiravir and raltegravir retention times were respectively 4.5 minutes and 14.5 minutes.

Intraday and interday reproducibility of the method was assessed using three levels of concentration: 20, 200 and 400 µg/mL. Both intra and interday coefficients of variation were below 15% for these three levels of concentration (see table below).

S-Table. Quality control (QC) of the assay technique

|  | QC 20 µg/mL | ​QC 200 µg/mL | ​QC 400 µg/mL |
| --- | --- | --- | --- |
| Interday Accuracy (%) | ​6.25 | ​4.33 | ​3.49 |
| ​Interday CV (%) | ​12.7 | ​12.92 | ​6.5 |
| ​Intraday Accuracy (%) | ​6.42 | ​3.97 | ​11.83 |
| Intraday CV (%) | ​8.85 | ​8.17 | ​2.24 |

**References:**

1. Madelain V, Guedj J, Mentré F, Nguyen THT, Jacquot F, Oestereich L, et al. Favipiravir pharmacokinetics in non-human primates: insights for future efficacy studies of haemorrhagic fever viruses. Antimicrob Agents Chemother. 2016; AAC.01305-16. doi:10.1128/AAC.01305-16

2. Japanese Pharmaceuticals and Medical Devices Agency (PMDA). Report on the Deliberation Results (English version) [Internet]. 4 Mar 2014 [cited 31 May 2016]. Available: https://www.pmda.go.jp/files/000210319.pdf
