# Supplementary material for: Favipiravir pharmacokinetics in Ebola-Infected patients of the JIKI trial reveals concentrations lower than targeted
Source: PLoS Negl Trop Dis. 2017 Feb 23;11(2):e0005389. doi: 10.1371/journal.pntd.0005389 (PMC5340401; doi:10.1371/journal.pntd.0005389)
Supplement: S2 Text — (DOCX) [file pntd.0005389.s002.docx]

**S2 Text**

**Impact of inactivation procedure on plasma favipiravir concentration**

EBOV is classified as a biohazard level 4 virus. Due to logistical constraints, favipiravir plasma concentrations cannot be assayed inside the INSERM BSL4 laboratory. The French regulatory agency (ANSM) imposed plasma samples from EBOV infected samples to be inactivated before being transferred to another INSERM laboratory where favipiravir concentration can be assayed. As favipiravir is stable at high temperature, the chosen inactivation strategy was to heat plasma sample at 60°C for one hour, leading to the loss of infectivity of viral particles present in the sample.

The evaluation of the heating procedure on plasma favipiravir concentrations was made using plasma samples collected in a pharmacokinetic study conducted in uninfected nonhuman primates given the dose equivalent to those used in humans in the JIKI trial in order to prepare for the quantification of favipiravir concentrations in infected plasma collected in humans and animals. Assays were therefore performed in duplicate in this study, after heating or not, in order to assess impact of this procedure on plasma favipiravir concentrations measurement.

*Material and methods*

Two hundred plasma samples obtained in 5 female cynomolgus macaques from Mauritius Island receiving a loading dose of 200 mg/kg followed by a maintenance dose of 100 mg/kg, every 12 hours by short infusion of 10 min (Reaction 1 study - approved by French research ministry, conducted by the companies SILABE and Eurofin/ADME bioanalyses in France on behalf of the academic European Reaction consortium [1]) were used to evaluate the inactivation assessment. Each plasma sample, for each sample time and each monkey, was divided into 2 aliquots. One was heated for 1 hour at 60 °C, before it was frozen at – 20 °C; the second one was directly frozen at – 20°C. Both were then assayed with the same analytical protocol using high performance liquid chromatography (Kromasil C18) coupled to tandem mass spectrometry detection (API4000).

Assessment of the agreement of the concentrations from the two aliquots was performed computing heated and non-heated plasma concentrations correlation, absolute difference and relative difference for each sample, as:

Absolute difference = Heated plasma concentration – non heated plasma concentrations;

Relative difference = (Heated plasma concentration – non-heated plasma concentrations)/ non-heated plasma concentrations.

Median, (minimum; maximum), of absolute difference and relative difference were calculated. Data below the limit of quantification (LOQ) were excluded from the analysis and reported separately.

*Results*
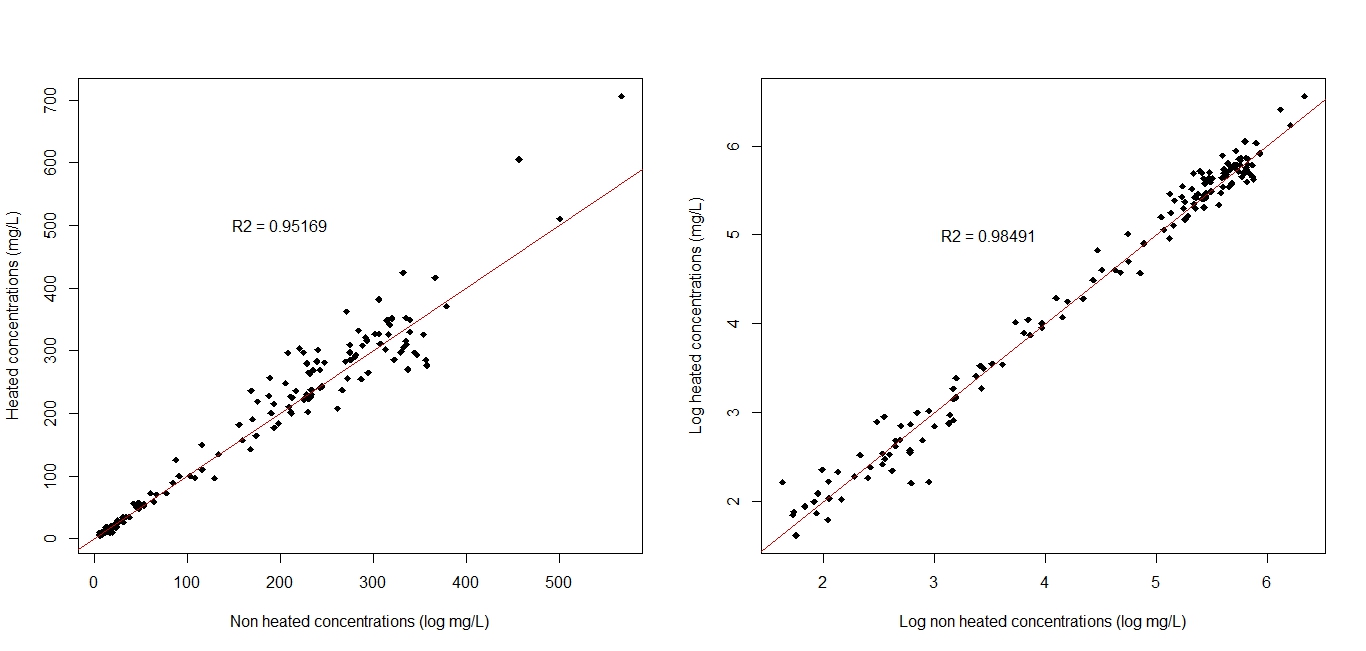


**Fig A.** Favipiravir natural (left) and logarithmic (right) concentrations assayed on non-heated plasma plotted vs ones assayed on heated plasma. Red line represents identity line.


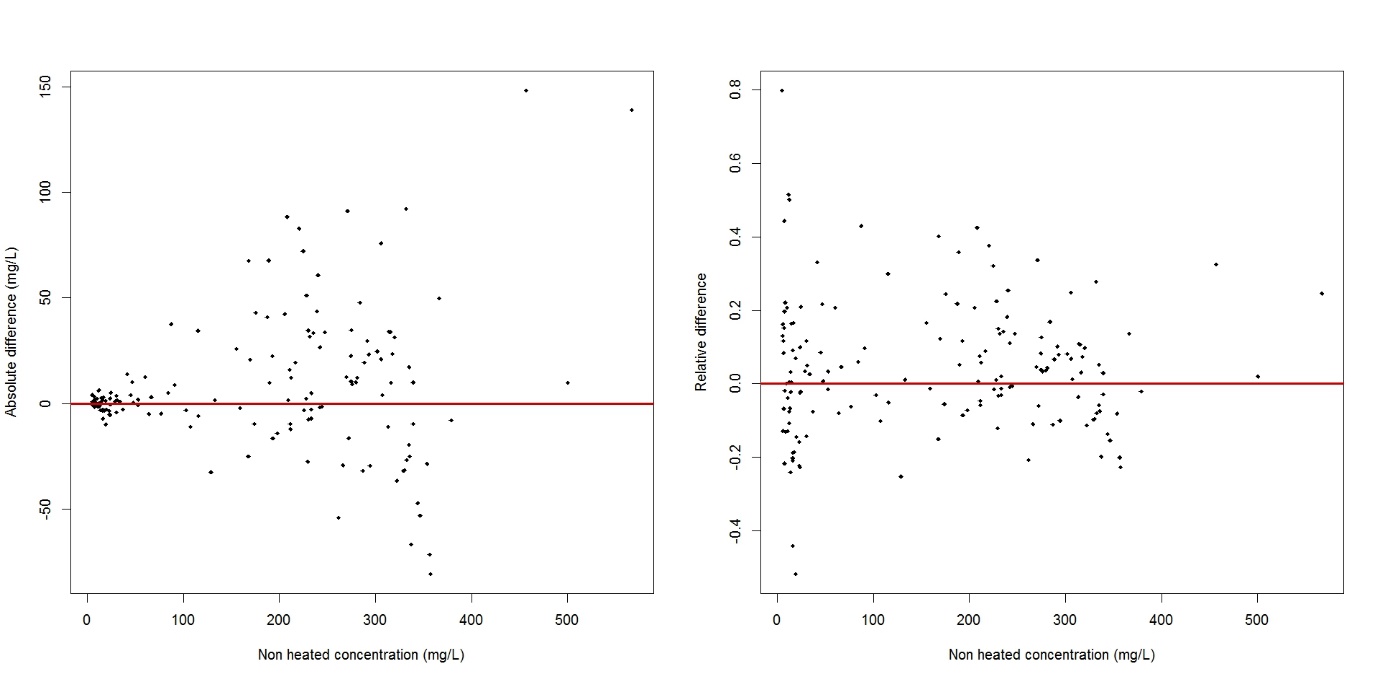


**Fig B.** Absolute and relative differences of favipiravir concentrations plotted versus non-heated concentration.

**Fig A** and **B** show good agreement between the two pre analytical processes. Relative concentration differences are uniformly scattered on both sides of the 0 line, in favour of absence of difference. The computed median absolute difference was 1.33 (-81.01; 148.47) mg/L, but we can see on **Fig B** that absolute differences are lower for the lowest level of concentration. Median of relative difference was found to 3.1% (-51.9%; 79.9%), confirming good agreement. For the concentrations below the LOQ, discordance existed for 5 samples, and 36 showed agreement.

In conclusion, the described inactivation protocol appears to have no relevant systematic impact on the measurement of favipiravir concentrations, and was used to assess plasma concentrations from infected samples.

**Reference:**

1. Madelain V, Guedj J, Mentré F, Nguyen THT, Jacquot F, Oestereich L, et al. Favipiravir pharmacokinetics in non-human primates: insights for future efficacy studies of haemorrhagic fever viruses. Antimicrob Agents Chemother. 2016; AAC.01305-16. doi:10.1128/AAC.01305-16
