# Supplementary material for: Favipiravir pharmacokinetics in Ebola-Infected patients of the JIKI trial reveals concentrations lower than targeted
Source: PLoS Negl Trop Dis. 2017 Feb 23;11(2):e0005389. doi: 10.1371/journal.pntd.0005389 (PMC5340401; doi:10.1371/journal.pntd.0005389)
Supplement: S3 Text — (DOCX) [file pntd.0005389.s003.docx]

**S3 Text**

**Comparison of plasma and serum concentrations**

We compared the concentrations obtained in plasma and serum at Day-2 and Day-4 after treatment in the following **Table S** and **Fig S**. Clearly the drug concentrations in plasma or serum were largely comparable, both at Day-2 (median value of 48.20 and 44.0 µg/mL in plasma and serum, respectively, p=0.86) and Day-4 (median value of 27.1 and 20.9 µg/mL in plasma and serum, respectively, p=0.35). In particular, the same clear trend towards a reduction in drug concentration between Day-2 and Day-4 was observed in both plasma and serum (**Fig S**).


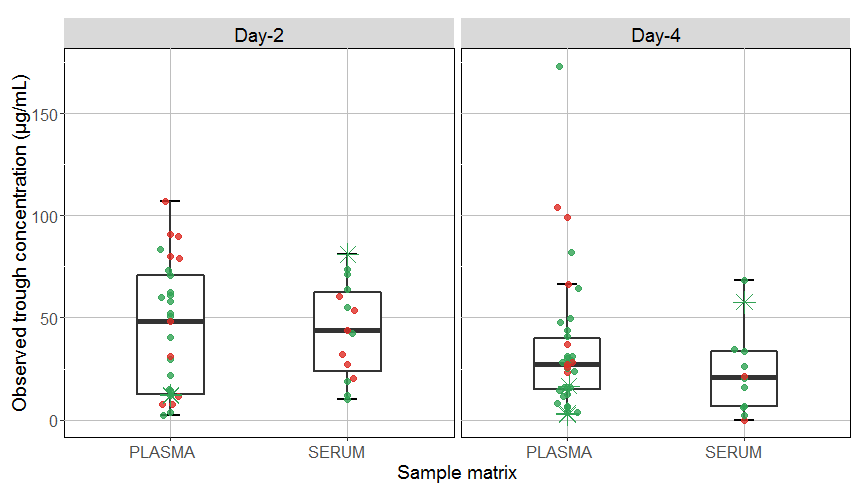


**Fig S**. Comparison of plasma and serum concentrations of favipiravir obtained at Day-2 and Day-4 after treatment initiation. Red points represent concentrations measured in patients who died during the trial, green points represent concentrations measured in those who survived. Concentrations obtained in patients receiving adult dose or weighted-based dose are presented in circles and stars, respectively.

**Table S**. Summary of favipiravir concentrations obtained in plasma and serum at Day-2 and Day-4.

|  | **Plasma**  **Median (min-max), number of observations** | **Serum**  **Median (min-max), number of observations** | **p-value** |
| --- | --- | --- | --- |
| **Day-2** | 48.20 (2.33-106.90), n=29 | 44.00 (10.30-81.00), n=15 | 0.86 |
| **Day-4** | 27.10 (2.70-173.20), n=38 | 20.85 (0.00-68.40), n=12 | 0.35 |
