# Supplementary figures and images for: Favipiravir pharmacokinetics in Ebola-Infected patients of the JIKI trial reveals concentrations lower than targeted
Source: PLoS Negl Trop Dis. 2017 Feb 23;11(2):e0005389. doi: 10.1371/journal.pntd.0005389 (PMC5340401; doi:10.1371/journal.pntd.0005389)

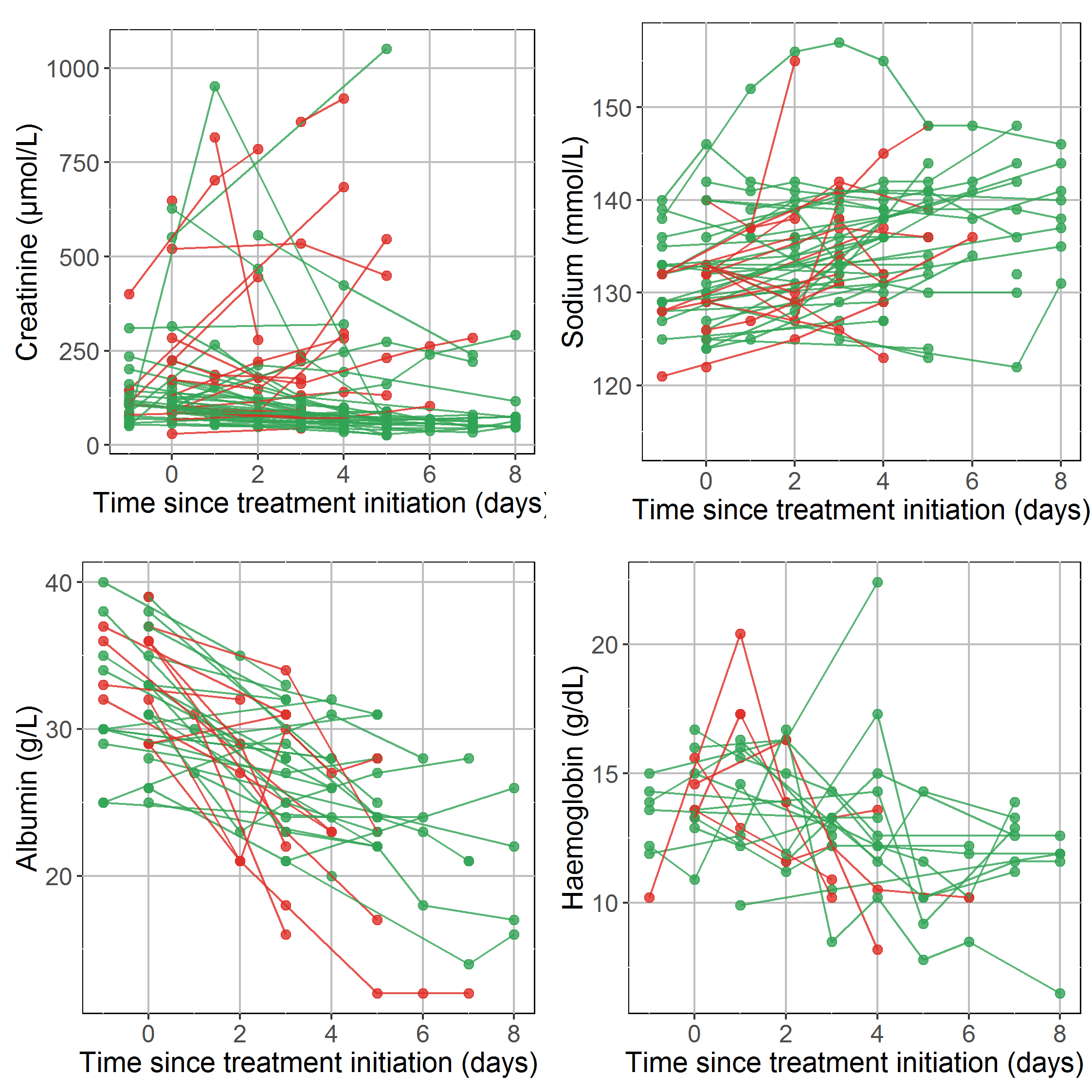

Supplement: S1 Fig — Red points represent concentrations measured in patients who died during the trial, green points represent concentrations measured in those who survived (n = 66, 62, 43, and 23 for creatinine, sodium, albumin, haemoglobin, respectively). (TIFF) [file pntd.0005389.s004.tiff]

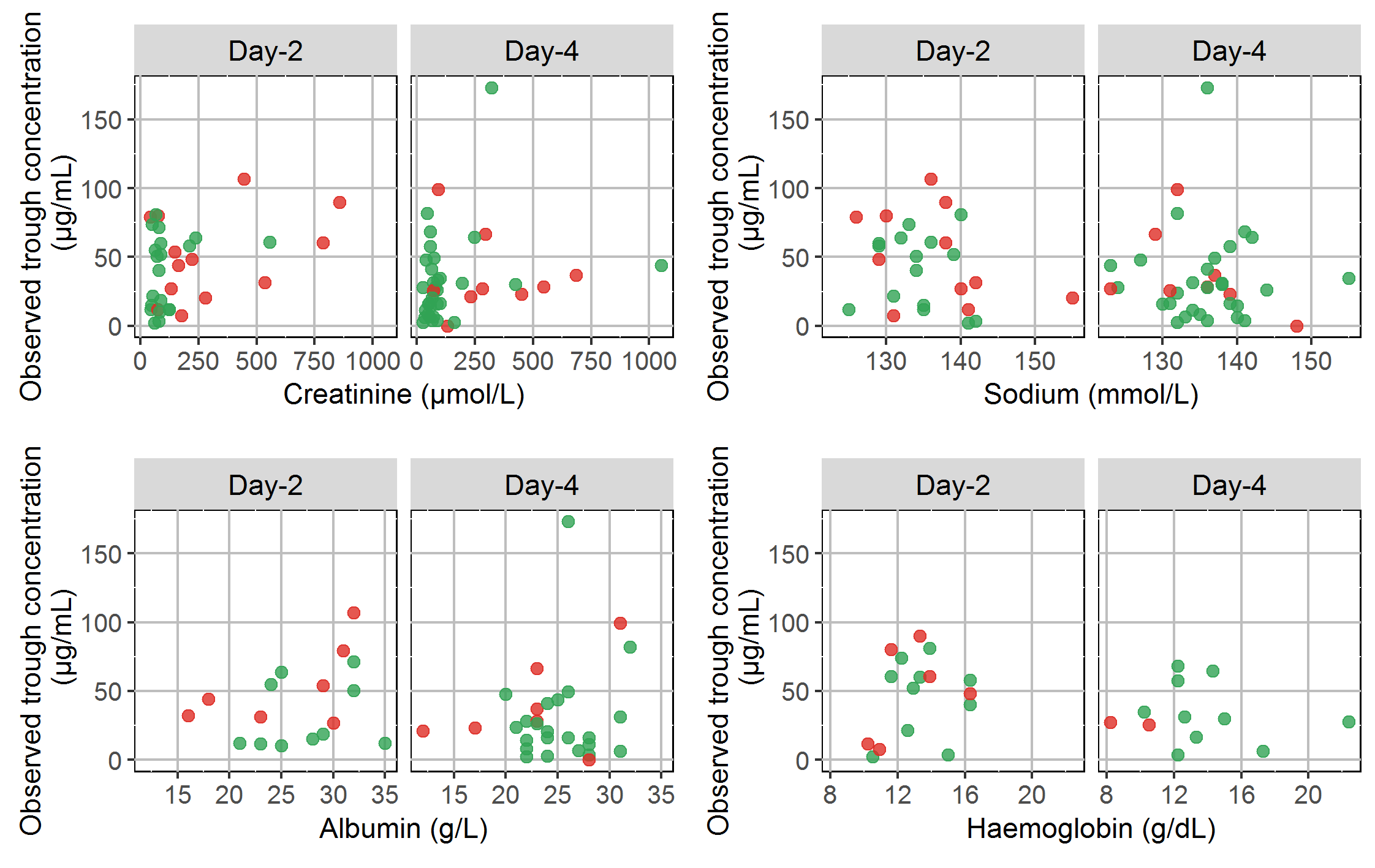

Supplement: S2 Fig — Red points represent concentrations measured in patients who died during the trial, green points represent concentrations measured in those who survived. At Day-2, number of patients included in these graphs was 33, 26, 17 and 16 for creatinine, sodium, albumin and haemoglobin, respectively. At Day-4, number of patients included these graphs was 42, 36, 29 and 12 for creatinine, sodium, albumin and haemoglobin, respectively. (TIFF) [file pntd.0005389.s005.tiff]
